# COVID-19: A New Horizon in Congenital Heart Diseases

**DOI:** 10.3389/fped.2021.582043

**Published:** 2021-12-08

**Authors:** Ehsan Aghaei Moghadam, Shabnam Mohammadzadeh, Roya Sattarzadeh Badkoubeh, Azin Ghamari, Ali Rabbani, Ali Mohebbi, Aliakbar Zeinaloo, Mahmoudreza Ashrafi, Niyoosha Kamran, Paniz Masoominasab, Zahra Mahmoudi, Asma Zamani Mehryan, Mohammad Reza Mirzaaghayan

**Affiliations:** ^1^Growth and Development Research Center, Tehran University of Medical Science, Tehran, Iran; ^2^Department of Cardiology, Tehran University of Medical Sciences, Tehran, Iran; ^3^Pediatric Department, Tehran University of Medical Sciences, Tehran, Iran

**Keywords:** congenital heart disease, COVID-19, pandemic, ACE inhibitor, cardiac surgery

## Abstract

**Objective:** Previous studies have demonstrated that both children and adult patients with a history of congenital heart disease (CHD) are at high risk for coronavirus disease 2019 (COVID-19) infection. This study investigates the status of COVID-19 infection among children undergoing surgical repair within the past 2 years.

**Methods:** All alive patients operated on in a tertiary referral center between March 2018 and March 2020 were recruited in the present study. Detailed demographics, past medical and surgical history, and physical examination were reviewed for each patient. During the COVID-19 pandemic, data regarding the patient's status were collected by telephone survey from April 15 to April 30, 2020.

**Results:** A total number of 210 patients are analyzed in this study. Participants' median age was 21.59 months [interquartile range (IQR) = 12–54.67], and 125 (59.5%) were female. The median interval between surgery and COVID-19 assessment was 305 days (IQR = 215–400). In addition, 67 (32%) patients used angiotensin receptor blocker (ARB)/angiotensin-converting enzyme (ACE) inhibitor (spironolactone and/or captopril). Sixteen patients (7.6%) were symptomatic and had positive chest CT results and/or RT-PCR compared to the previously reported prevalence of COVID-19 among the pediatric population (2.4% of children with <18 years of age); the prevalence of COVID-19 among the patients operated on due to CHD in the present study was significantly higher (*p* = 0.00012). Two patients were admitted to the intensive care unit (ICU); one patient was discharged 2 weeks later with acceptable status, and one patient died 2 days after ICU admission due to cardiac and respiratory arrest and myocarditis. The complexity of the underlying cardiac disorders was not different between patients with low risk (*p* = 0.522), suspicious patients (*p* = 0.920), and patients positive for COVID-19 (*p* = 0.234). The ARB/ACE inhibitor consumption was not associated with the COVID-19 infection [*p* = 0.527, crude odds ratio (OR) = 1.407, 95% CI = 0.489–4.052].

**Conclusion:** Children with a history of previous CHD surgery are more susceptible to infections, especially those infections with pulmonary involvements, as the lung involvement could cause worsening of the patient's condition by aggravating pulmonary hypertension. The results of the current study indicate that these patients are more prone to COVID-19 infection compared to the healthy children population.

## Introduction

Novel beta-coronavirus [coronavirus disease 2019 (COVID-19)], described first in December 2019, is a complex disease with various and sometimes confusing clinical manifestations (1). This infection is transmitted by human-to-human contact *via* droplets, contact, and entry *via* ocular tissue with high transmission rates (R_0_ = 2–3.8) (1–3). Fever, dry cough, and shortness of breath are the most common manifestations; abdominal pain, nausea, and diarrhea could occur (4, 5). Multiorgan failure and disseminated intravascular coagulation (DIC) are also presented as a result of the overall inflammatory response (2); furthermore, hypoxia deteriorates the end-organ dysfunction and precipitates death in critically ill cases (5). The heart is involved in this infection by several mechanisms: oxidative stress due to increased myocardial oxygen demand and decreased oxygen supply due to hypoxia caused by pulmonary involvement (6); inordinate immune–inflammatory response and cytokine storm (7); direct invasion of the virus into the cardiac myocyte (which is not proven by histology evaluations) (8). Reports from Wuhan and the Hubei region revealed that a positive past medical history for cardiovascular comorbidities puts patients with COVID-19 at a higher risk of morbidity and mortality (2, 9, 10). Previous studies have demonstrated that both children and adult patients with a history of congenital heart diseases (CHDs) are at high risk for COVID-19 infection (6, 11). This study investigates the status of COVID-19 infection among children undergoing surgical repair within the past 2 years.

## Materials and Methods

### Study Design

All alive patients operated on in a tertiary referral center between March 2018 and March 2020 were recruited in the present study. Surgeries were performed by one surgeon in a tertiary referral center. All surgical notes and medical records of patients are reviewed, and patients' contact numbers and addresses were extracted using our database. Detailed demographics, past medical and surgical history, and physical examination were reviewed for each patient. During the COVID-19 pandemic, data regarding the patient's status were collected by telephone survey from April 15 to April 30, 2020. Two trained interviewers recorded the patients' current situation, recent hospital admission, exposure level, and personal care. The presence of the following symptoms, including cough, fever, dyspnea, myalgia, sore throat, and/or fatigue in patients and their household members in the period from the first official announcement of COVID-19 infection in Iran until the time of phone interview was assessed. The patients were considered at low risk for COVID-19 if none of those mentioned earlier symptoms existed, suspicious if one or two symptoms existed for <3 days, and positive if positive clinical evaluations of COVID-19 by infectious consultation exist plus the positive chest CT results and/or positive reverse transcriptase-polymerase chain reaction (RT-PCR) of the nasopharyngeal swab was in favor of COVID-19. COVID-19 pneumonia was considered positive in the case following features that were found in the CT scan: the presence of ground-glass opacity (GGO) mainly in the peripheral and posterior lungs that did not spare the subpleural regions, consolidation, GGO with consolidation, or interlobular septal thickening (12).

All the patients were asked to report any new symptoms or definite COVID-19 infections within 14 days after the phone interview with a hotline that served as a connection. The underlying cardiac defect classification was performed using the Adult Congenital Heart Disease Anatomic and Physiological (ACHD AP) classification (13) as simple, moderate, and severe complexity.

### Preventive Measure Assessments

Five levels of preventive measures were defined as follows:

Level 0: No care, no change in daily routine activity and the patient's attitude and the household.
Level 1: Mild care, mildly changed daily routine activity [occasional handwashing and wearing the mask, but not precisely based on WHO advice (14)].
Level 2: Restricted care for the patient (protective measures strictly based on WHO advice for the public) with adherence to social distancing protocol in the community.
Level 3: Restricted care plus adherence to social distancing plus adherence to governmental “stay at home” advice strictly (quarantine of the patient).
Level 4: Restricted care plus all family members' adherence to social distancing and staying at home (quarantine the whole family members and household).

### Exposure Level

Level 0: No history of contact with a symptomatic patient or confirmed COVID-19 case.
Level 1: History of contact with a symptomatic patient or household (highly suggestive of COVID-19 infection without a confirmed COVID-19 test).
Level 2: Presence of confirmed COVID-19 case in the household.

### Statistical Analysis

All data were analyzed using SPSS version 22. Results are presented as the number (percent), mean (±standard deviation), and mean [interquartile range (IQR)]. The normal distribution was tested using the Kolmogorov–Smirnov test. For parametric variables, Student's *t*-test was applied; for non-parametric ones, Mann–Whitney test was applied. The chi-square test was used to evaluate relationships between categorical variables. The association between variables was assessed using a linear regression test. A *p*-value below 0.05 was considered statistically significant.

### Ethical Considerations

This study has been approved by the Research Deputy and the Ethics Committee of Tehran University of Medical Sciences and has been conducted according to the ethical standards laid down in the 1964 Declaration of Helsinki and all subsequent revisions.

## Results

A total of 230 patients were operated on, 20 patients were excluded from the study (five patients had died, and 15 were not available by phone call). Participants' median age was 21.59 months (IQR = 12–54.67), and 125 (59.5%) were female. The median interval between surgery and COVID-19 assessment was 305 days (IQR = 215–400). Since the official announcement of COVID-19 infection, hospital admission history due to reasons other than infection was positive in 10 (4.8%) patients. Pacemaker insertion, due to complete heart block, was performed in five (2.4%) patients. Moreover, 67 (32%) patients used angiotensin receptor blocker (ARB)/angiotensin-converting enzyme (ACE) inhibitor (spironolactone and/or captopril). Concomitant medical disorder, other demographic data, past medical and drug history, and underlying cardiac disorder complexity are summarized in Table 1.

**Table 1:** Demographic data and the complexity of underlying cardiac disease among 210 patients.

**Variable**	**Information**
Mean age at the time of surgery (±SD)	27.89 (±34.94) months
**Average weight (±SD)**	
At the time of survey	12.66 (±7.8) kg
At the time of surgery	9.9 (±7.9) kg
**Concomitant medical disorder, number (%)**	
Hypothyroidism	9 (4.28%)
Seizure	4 (1.9%)
Esophageal atresia	2 (0.95%)
Neurodevelopmental delay	5 (2.38%)
**Genetic disorder, number (%)**	
Down syndrome	2 (0.95%)
**Drug consumption history, number (%)**	
Sildenafil	8 (3.8%)
Furosemide	62 (29.5%)
Spironolactone	53 (25.2%)
Aspirin	17 (8.3%)
Captopril	14 (6.8%)
Corticosteroid	2 (0.95%)
**Cardiac abnormality complexity**	
Simple complexity	42 (20%)
Moderate complexity	113 (53.8%)
Severe complexity	55 (26.2%)

Sixteen patients (7.6%) were symptomatic and had positive chest CT results and/or RT-PCR (Table 2). The prevalence of COVID-19 among the patients operated on due to CHD in the present study was significantly higher than that of the normal population (*p* = 0.00012). Typical chest CT, besides the highly suggestive clinical status evaluated by infectious disease experts, was detected for 13 patients, positive RT-PCR for two, and both of them for one patient. Among patients positive for COVID-19, clinical symptoms included fever in eight (50%), diarrhea in four (25%), dyspnea in one (6.25%), dry cough in five (31.25%), and myalgia in one (6.25%) patient. Two patients were admitted to the intensive care unit (ICU). One patient was discharged 2 weeks later with an acceptable status. One patient died 2 days after ICU admission due to cardiac and respiratory arrest and myocarditis unresponsive to cardiopulmonary resuscitation. The COVID-19 infection had no significant association with recent hospital admission (*p* = 0.152, crude OR = 3.321, 95% CI = 0.643–17.156) and previous history of hypothyroidism (*p* = 0.689, crude OR = 0.645, 95% CI = 0.076–5.508) and pacemaker insertion (*p* = 0.999).

**Table 2:** Coronavirus disease 2019 (COVID-19) status, preventive measures, and exposure levels of 210 patients.

**COVID-19 status of the patient**	
Low risk	177 (84.3%)
Suspicious	17 (8.1%)
Positive	16 (7.6%)
**Preventive measure assessments**	
Level 0	18 (8.6%)
Level 1	34 (16.2%)
Level 2	29 (13.8%)
Level 3	27 (12.9%)
Level 4	102 (48.6%)
**Exposure level**	
Level 0	195 (92.9%)
Level 1	12 (5.7%)
Level 2	3 (1.4%)

The rate of positive COVID-19 was not different between boys and girls (*p* = 1.000). The complexity of the underlying cardiac disorders was not different between patients with low risk (*p* = 0.522), suspicious patients (*p* = 0.920), and patients positive for COVID-19 (*p* = 0.234). The ARB/ACE inhibitor consumption was not associated with the COVID-19 infection (*p* = 0.527, crude OR = 1.407, 95% CI = 0.489–4.052). COVID-19 had no statistically significant association with age (*p* = 0.335, crude OR = −0.67) and complexity of the underlying CHD (*p* = 0.250, crude OR = 0.080). The exposure level was not associated with COVID-19 infection (*p* = 0.122, crude OR = 0.143, 95% CI = 0.12–1.68).

## Discussion

CHDs are the most common types of congenital disability. As medical care, surgical techniques and palliative treatments have improved; babies with a CHD survive more than they did before. Approximately 25% of these patients have critical conditions who need immediate intervention (15). Both children and adult patients with a history of CHDs are at high risk for complications in the COVID-19 outbreak (6, 11); these patients are more prone to cardiovascular disorders and infections (16), especially in case other comorbidities, such as pulmonary hypertension and heart failure, exist (6, 11, 16). Based on the ACHD AP stage classification, patients with complex heart lesions, including all forms of cyanotic CHDs and tricuspid atresia and single ventricles, are better considered at high risk for COVID-19 infection, as their functional reserve is decreased (13, 17). This could be extended to the pediatric population with CHD as well; however, the results of the current study demonstrated that the complexity of disease was not associated with the COVID-19 disease. The severity of the infection is more highlighted in patients with syndromes and asplenia, which are positively associated with CHDs, due to disturbed immunity. Up to the present, several studies have described the occurrence of myocarditis in healthy individuals with COVID-19 (18–21). In our series of patients, one patient died due to COVID-19-induced myocarditis.

It has been shown that children, compared to adults, have a lower risk of being infected with COVID-19. Less than 1% of the pediatric population younger than 10 years (22) and 2.4% younger than 18 years (23) are infected with COVID-19. The prevalence of COVID-19 disease in the present study was significantly higher than that in the normal population (*p* = 0.00012); it is important to take greater care to prevent COVID-19 in these patients; preventive care includes handwashing, social distancing, use of masks and other personal protective equipment, and cleaning and disinfecting commonly touched surfaces (24).

**Figure 1:**
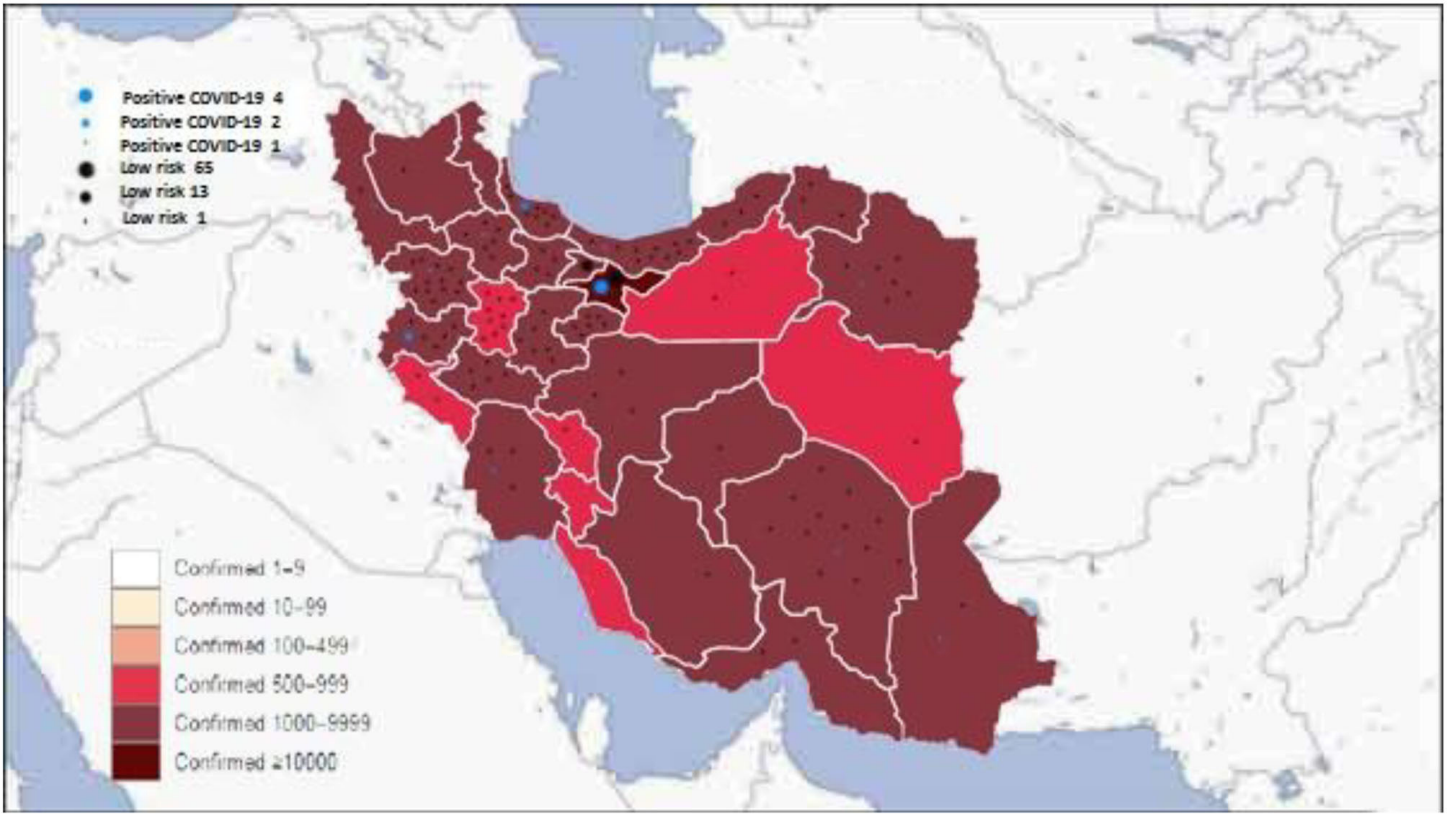
Distribution of the confirmed coronavirus disease 2019 (COVID-19) in Iran in each province and our patients' COVID-19 status.

Compared to adults, COVID-19 in pediatrics has been less studied. The incidence of COVID-19 varies by location and likely depends on several factors, including population density and demographics, the extent of testing and reporting, and the timing of mitigation strategies. Most of our patients were from the capital of Iran, Tehran (69 out of 210) (Figure 1), and 25% of positive patients were from Tehran, where the confirmed cases were highest compared to other provinces (Figure 1). A report of 72,314 COVID-19 patients from the Chinese Centers for Disease Control and Prevention indicated that 1% of cases were younger than 9 years (25). Dong et al. (26) assessed 2,143 pediatrics with COVID-19; 731 patients had confirmed COVID-19 by real-time PCR, and one patient died due to it. They indicated that most of these cases have mild-to-moderate severity, and 10% had a severe form of the disease. They found that younger children, especially infant ones, are more susceptible to a severe form of COVID-19 (26); however, our study's results showed no significant association between age and COVID-19 disease. Most patients with CHDs are diagnosed and operated on during infancy; therefore, these patients are often young. The combination of CHDs and young age is a warning sign of a more severe form of COVID-19 in these patients. Another study evaluated and tested 1,391 children younger than 16 years; 12.3% were positive for COVID-19, 1.3% needed ICU mechanical ventilation, and a 10-month-old patient with a history of intussusception died due to COVID-19 infection (27).

A multicenter nationwide study in Italy was performed by Sabatino et al. (28) to evaluate the clinical characteristics and outcomes in patients with CHD and COVID19. They found that the cardiovascular complications among patients with confirmed COVID-19 infection comprise heart failure (9%), palpitations/arrhythmia (3%), stroke/transient ischemic attack (3%), and pulmonary hypertension (3%) (28). Despite previous assumptions declaring a more severe form of infection in patients with CHDs (6, 11, 16, 26, 27), they observed a mild clinical course of COVID-19 in CHD patients (28). In our patients, two patients (out of 16) with COVID-19 were admitted to the ICU (a severe form of COVID-19), and the others had mild to moderate forms (14 out of 16).

## Conclusion

Children with a history of previous CHD surgery are more susceptible to infections; these patients need more care to prevent infection occurrence, especially those infections with pulmonary involvement, as lung involvement could result in worsening of the patient's condition by aggravating pulmonary hypertension. The results of the current study indicate that these patients are more prone to COVID-19 infection compared to the healthy children population.

### Limitations

The present study has limitations to be acknowledged. Small sample size is the main limitation; furthermore, the patients contacted us if the suspicious symptoms emerged until 2 weeks after the telephone contact—we did not follow the patients. COVID-19 was not confirmed in suspicious patients due to limited access to RT-PCR and other laboratory tests in some regions (such as rural regions) and great distance to the medical centers. Conducting a prospective multicenter study with an acceptable follow-up period and providing accessibility for testing and confirming COVID-19 for all suspicious patients around the country are recommended.

## Data Availability Statement

The original contributions presented in the study are included in the article/supplementary material, further inquiries can be directed to the corresponding author/s.

## Ethics Statement

This study has been approved by the Research Deputy and the Ethics Committee of Tehran University of Medical Sciences and has been conducted according to the ethical standards laid down in the 1964 Declaration of Helsinki and all subsequent revisions. Verbal consent was obtained from all participants' parents/legal guardians.

## Author Contributions

All authors listed have made a substantial, direct and intellectual contribution to the work, and approved it for publication.

## Conflict of Interest

The authors declare that the research was conducted in the absence of any commercial or financial relationships that could be construed as a potential conflict of interest.

## Publisher's Note

All claims expressed in this article are solely those of the authors and do not necessarily represent those of their affiliated organizations, or those of the publisher, the editors and the reviewers. Any product that may be evaluated in this article, or claim that may be made by its manufacturer, is not guaranteed or endorsed by the publisher.
